# Extraskeletal osteosarcoma infiltrating pancreas, spleen, gastric, and left kidney: a case report

**DOI:** 10.1186/s13256-024-04608-x

**Published:** 2024-07-10

**Authors:** Wifanto Saditya Jeo, Shintia Christina, Nathaniel Jason Zacharia, Khalikul Razi

**Affiliations:** 1 Digestive Surgery Division, Surgery Department, Dr. Cipto Mangunkusumo National Central General Hospital, Jakarta, Indonesia; 2 Pathological Anatomy Department, Siloam Kebon Jeruk Hospital, Jakarta, Indonesia; 3 Digestive Surgery Division, Surgery Department, Dr. Zainoel Abidin General Hospital, Banda Aceh, Indonesia

**Keywords:** Extraskeletal osteosarcoma, ESOS, Diagnosis, Rare malignancy

## Abstract

**Background:**

Extraskeletal osteosarcoma is an extremely rare malignancy that accounts for 1% of soft tissue sarcoma and 4.3% of all osteosarcoma. Extraskeletal osteosarcoma can develop in a patient between the ages of 48 and 60 years. The incidence of extraskeletal osteosarcoma is slightly higher in male patients than in females.

**Case presentation:**

A 50-year-old Caucasian male patient presented with a 6-month history of intermittent lower-left back pain that limits his activity. Prior ultrasonography and abdominal computed tomography scan showed a diagnosis of kidney stone and tumor in the lower-left abdomen. The computed tomography urography with contrast revealed a mass suspected as a left retroperitoneal malignant tumor. Hence, the tumor was resected through laparotomy and the patient continued with histopathological and immunohistochemistry examination with the result of extraskeletal osteosarcoma.

**Conclusion:**

Extraskeletal osteosarcoma presents diagnostic challenges requiring multimodal examination, including histological and immunohistochemistry analyses. This case underscores the aggressive nature and poor prognosis despite undergoing the current suggested treatment.

## Background

Extraskeletal osteosarcoma (ESOS) is a scarce osteosarcoma originating from mesenchymal cells. The uniqueness of this osteosarcoma is that it is located in soft tissue and other organs without any evidence of primary bone osteosarcoma. The lower extremities are the most common ESOS site. ESOS is an aggressive type of malignancy that accounts for 1% of soft tissue sarcoma and 4.3% of all osteosarcoma. This type of osteosarcoma can occur in patients between the ages of 48 and 60 years, with a slightly higher incidence in male patients than in females [1–5].

The most common sites of primary extraskeletal osteosarcoma are thigh soft tissue (46%), followed by upper extremities (20%), and retroperitoneum (17%). Despite that, ESOS can occur in any part of the body. About 4–13% of cases presented as secondary cancer owing to radiotherapy with 2–40 years length of therapy before cancer appears [2].

## Case presentation

The patient was a 50-year-old Caucasian male. He complained about lower-left back pain that intermittently lasted for 6 months. The pain severity got worse and limited his activity without any reported unexplained or sudden weight loss. His vital signs were within normal limits. The abdomen was flat without tenderness and had a typical bowel sound on physical examination. There was no palpable mass of the liver and spleen. From a prior abdominal ultrasonography (USG) examination, he was diagnosed with a left kidney stone. Afterward, an abdominal computed tomography (CT) scan showed a tumor in his lower left abdomen. The first hospital admission of the patient was in December 2021, and he underwent clinical treatment periodically until his time of death in September 2023 (Table 1).

**Table 1:** Timeline of the patient’s medical history

A 50-year-old male with left lower back pain diagnosed as extraskeletal osteosarcoma infiltrating the pancreas, spleen, gastric region, and kidney		
Intervention/examination	Date	Diagnostic significance/complaints/recommendations
	June 2021	Complaint: - Intermittent lower-left back pain - There is no unexplained or sudden weight loss
USG	24 December 2021	Diagnosis: kidney stone
CT—abdomen without contrast	9 January 2022	Diagnosis: tumor in the lower-left abdomen
CT—urography with contrast (Fig. 1)	10 January 2022	Diagnosis: suspect left retroperitoneal malignant tumor with most likely adrenal cancer or retroperitoneal sarcoma
First laparotomy	13 January 2022	Diagnosis presurgery: left retroperitoneal tumor-infiltrating pancreas, spleen, and left kidney Diagnosis post-surgery: retroperitoneal tumor-infiltrating pancreas, spleen, gastric region, and left kidney
Histopathological exam	15 January_,_ 2022	Diagnosis: high-grade sarcoma with a differential diagnosis of osteogenic extraskeletal sarcoma, extraskeletal chondrosarcoma, and MPNST with heterology element Recommendations: IHC examination
IHC exam	20 January 2022	Diagnosis: extraskeletal osteosarcoma
PET scan (First follow-up assessment)	21 February 2022	Diagnosis: no residual malignancy in the operating area of the upper-left abdominal cavity, no suspicion of the tumor in other organs, no metastasis to lymphatic nodes, and no metastasis to the lungs
CT—abdomen and pelvis CT—urography with contrast (second follow-up assessment; Fig. 5A)	7 September 2022	Diagnosis: there are multiple new nodules of very heterogeneous calcification at the left renal bed with multiple lymphadenopathy in paraaortic—aortocaval groups (intralesional calcification)
CT—abdomen and pelvis with contrast (third follow-up assessment; Fig. 5B)	24 January 2023	Diagnosis: larger and multiple calcified nodules in the renal bed and hepatogastric area with multiple lymphadenopathy calcifications
Second laparotomy	28 February 2023	Diagnosis pr-surgery: Recidive extraskeletal osteosarcoma Diagnosis postsurgery: Recidive extraskeletal osteosarcoma post radical re-excision
CT—abdomen and pelvis without contrast (Fig. 5C, D)	10 August 2023	Diagnosis: a new and large peritoneal carcinomatosis with a massive mass on the lung and pleural cavity
Last admission	21 September 2023	The patient died of multiple metastases

*USG* ultrasonography, *CT* computed tomography, *MPNST* malignant peripheral nerve sheath tumor, *IHC* immunohistochemical, *PET* positron emission tomography

**Fig. 1:**
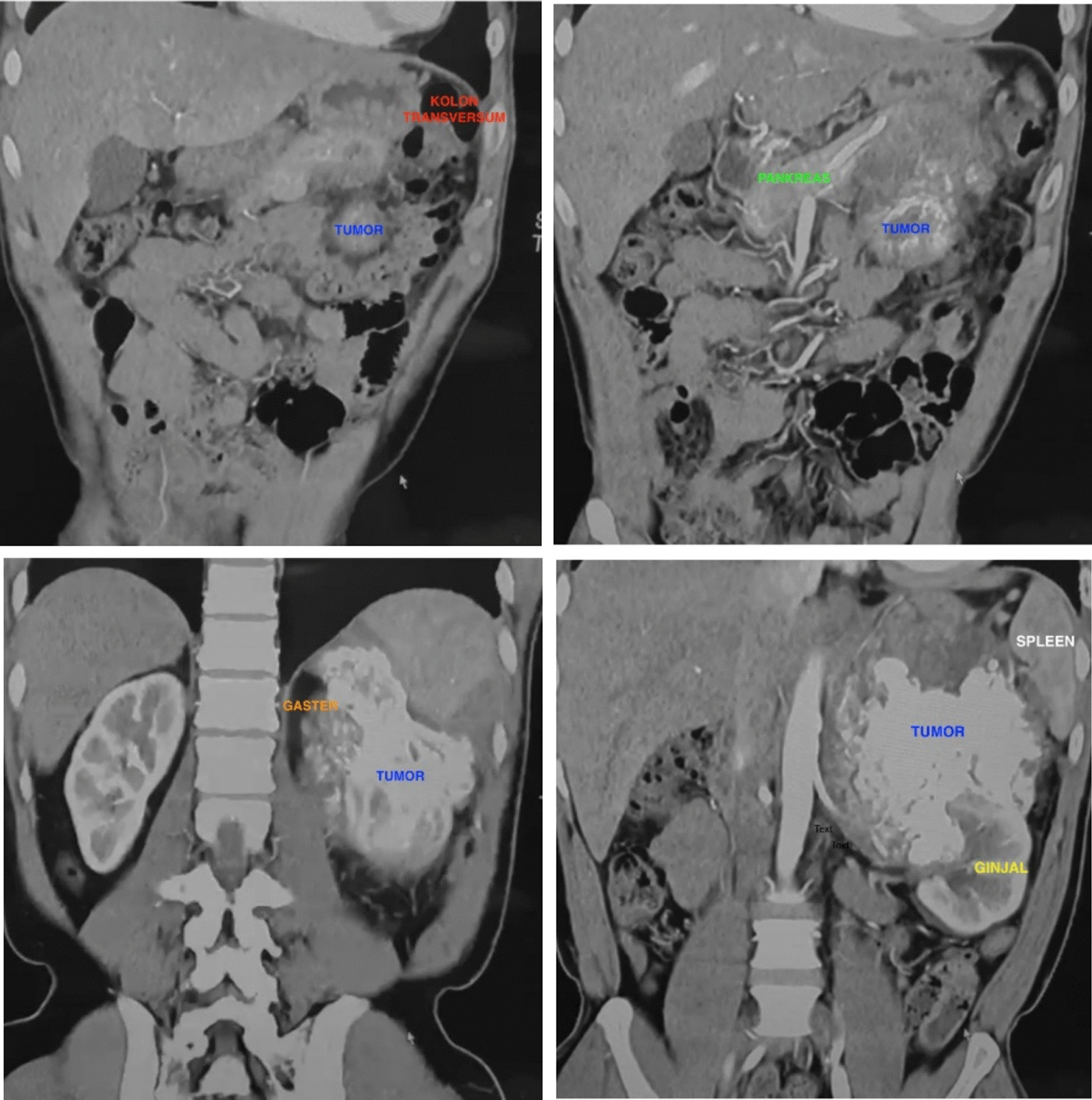
First computed tomography urography with contrast

The patient underwent a laboratory test and CT urography with contrast on two consecutive days. Laboratory test results were within normal range. CT urography with contrast revealed a prominent calcified solid mass infiltrating the surrounding structure, which is suspected as a left retroperitoneal malignant tumor (Fig. 1). By that time, the most likely diagnosis of the tumor was an adrenal cancer or retroperitoneal sarcoma.

**Fig. 2:**
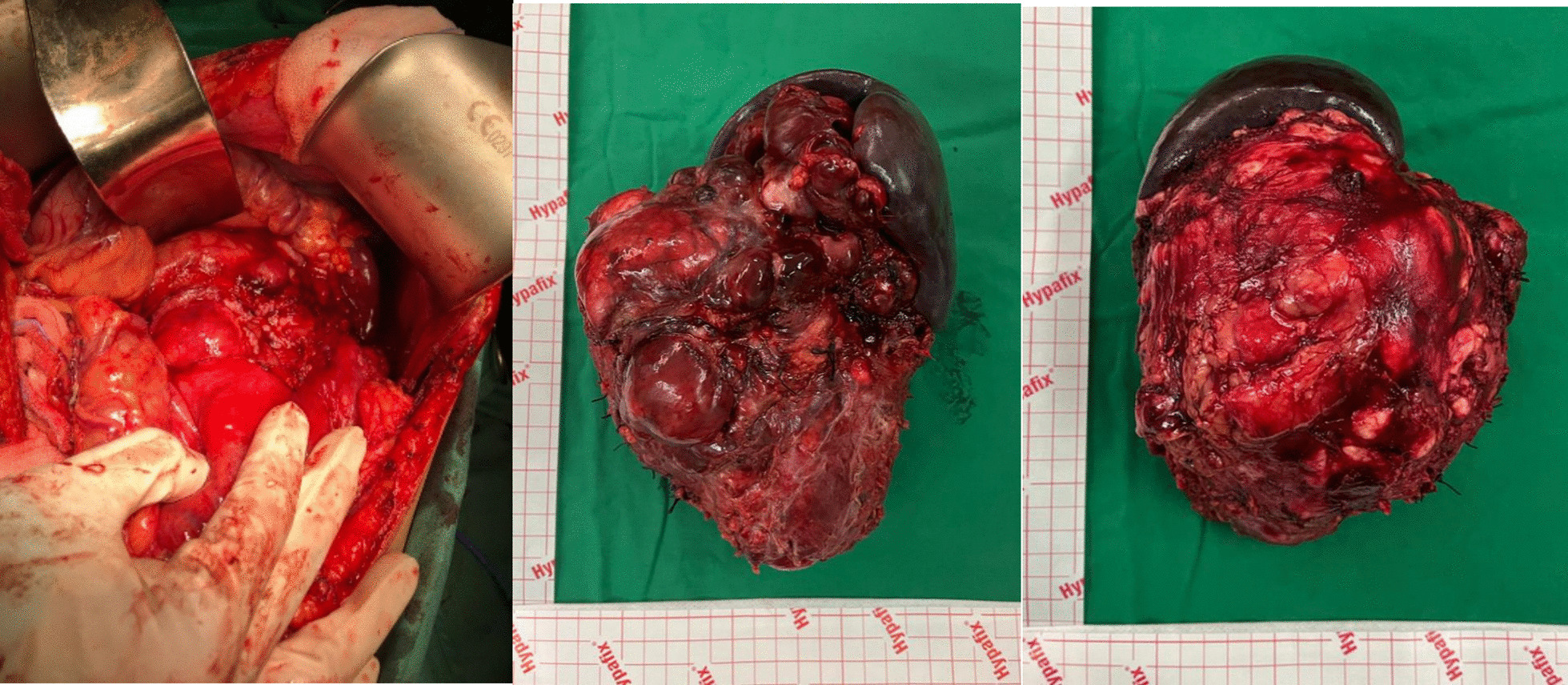
Intraoperative procedure and the tumor

To ensure the diagnosis and cure of the tumor, we performed laparotomy with a presurgery diagnosis of the left retroperitoneal tumor-infiltrating the pancreas, spleen, and left kidney. The procedures during laparotomy are tumor resection (Fig. 2), distal pancreatectomy, partial gastrectomy, splenectomy, and left nephrectomy. The diagnosis after surgery changed to left retroperitoneal tumor-infiltrating the pancreas, spleen, gastric region, and left kidney.

**Fig. 3:**
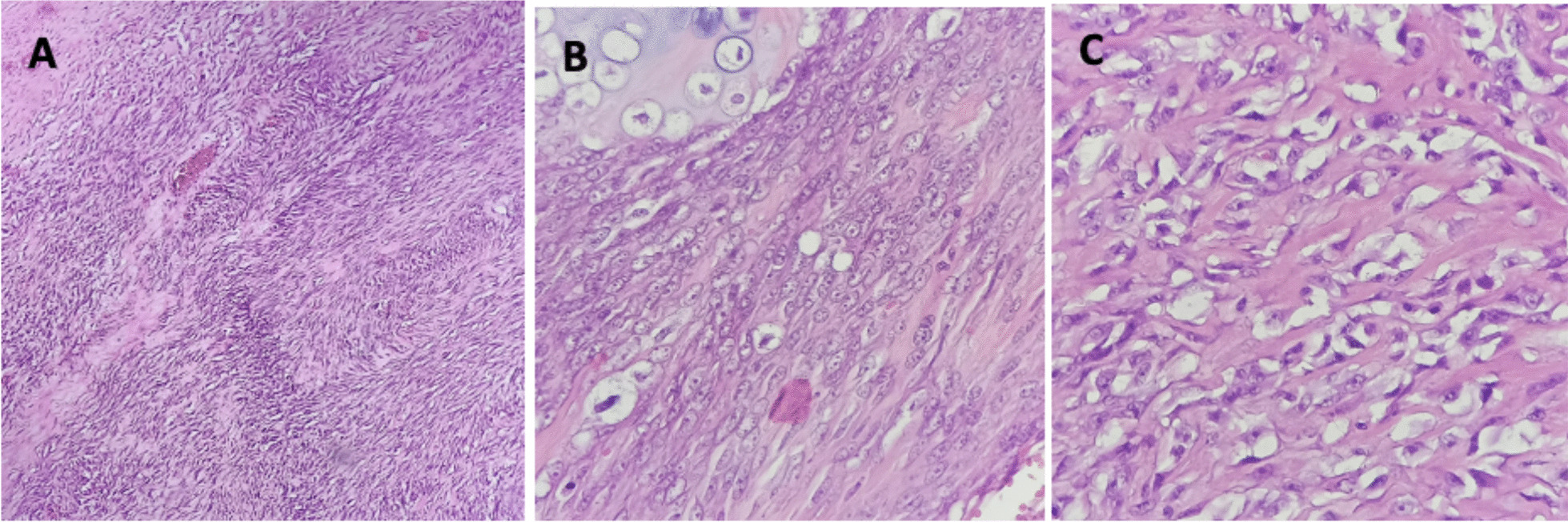
Histopathology of the tumor lesion. **A** Hematoxylin and eosin, 100 ×. Tumor cells are arranged in a fascicular growth pattern with high-grade spindle cells. **B** (Hematoxylin and eosin, 400 ×) Tumor cell nuclei are pleomorphic, coarse chromatin, and vesicular, some with prominent nucleoli. Some parts showed cartilage components. **C** (Hematoxylin and eosin, 400 ×) Lace-like osteoid with high-grade cells

**Fig. 4:**
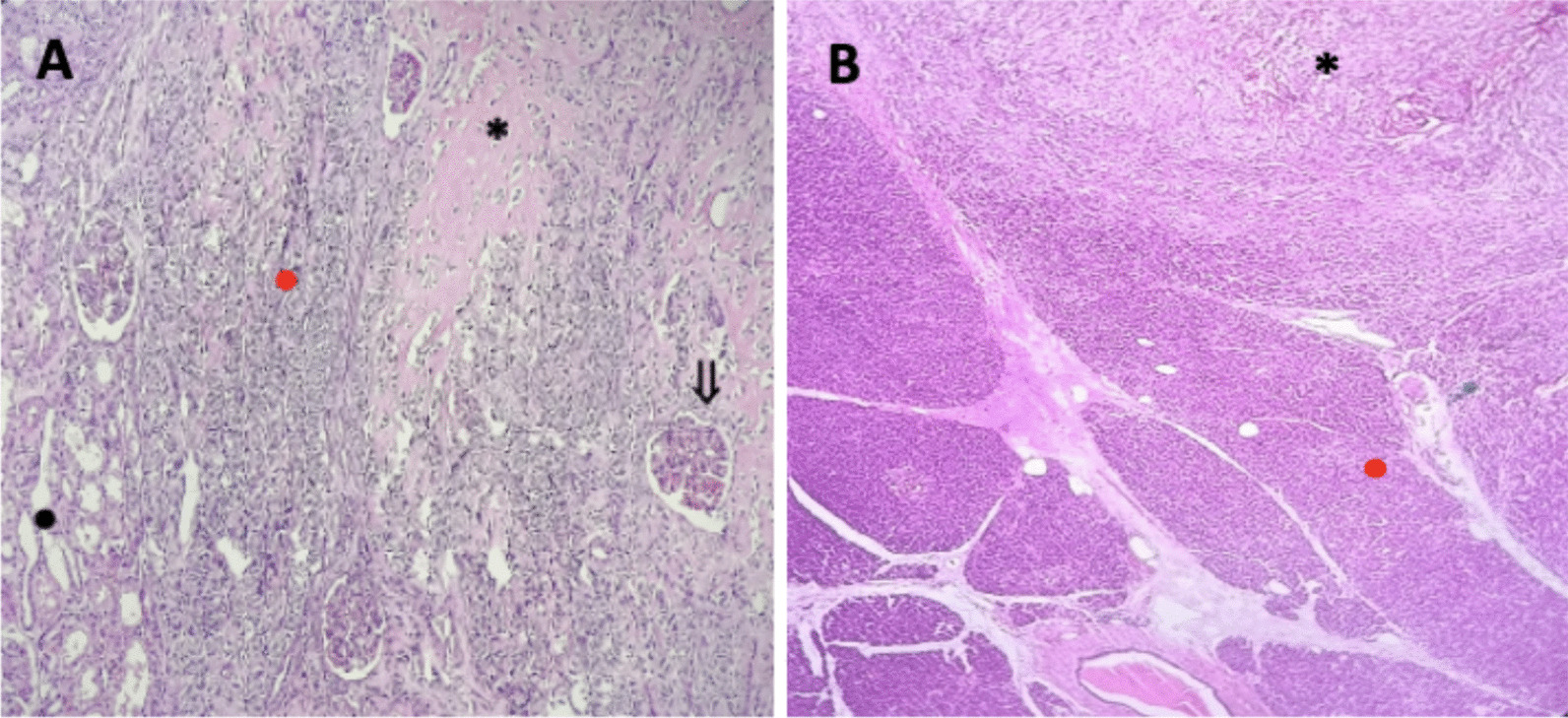
Histopathology of kidney and pancreas invasion. **A** Kidney, black dot: tubules, red dot: tumor cell, asterisk: osteoid, black arrow: glomerulus. **B** Pancreas, red dot: pancreas cell, asterix: tumor cell

After the laparotomy, the patient recovered well and was discharged from the hospital 7 days after surgery. A histopathological examination was done after surgery and showed a high-grade sarcoma with a differential diagnosis of osteogenic extraskeletal sarcoma, extraskeletal chondrosarcoma, and malignant peripheral nerve sheath tumor (MPNST) with heterology element (Fig. 3). The tumor invaded the kidney and pancreas but not the spleen (Fig. 4).

We performed an immunohistochemical (IHC) examination to confirm the diagnosis, and the following results were reported: positive for SATB2 and CD99 marker, partially positive of SMA and EMA marker, low partially positive of AR marker, negative of S100 marker, positive for 20–40% of Ki67 marker. The conclusion of the diagnosis from the IHC examination was extraskeletal osteosarcoma.

**Fig. 5:**
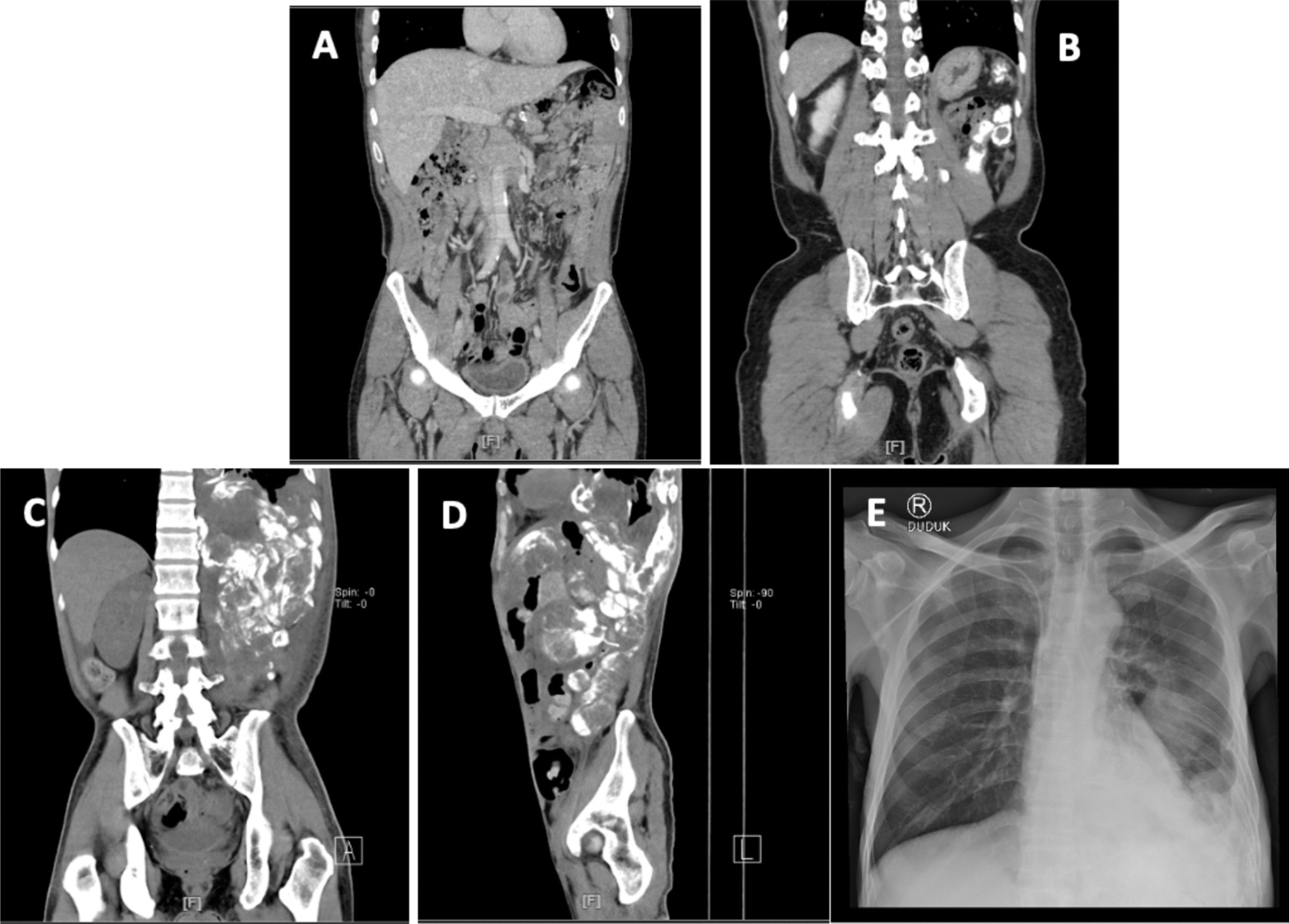
Routine scheduled imaging follow-up after surgery. **A** Computed tomography—abdomen and pelvis without contrast, coronal view (8 months). **B** Computed tomography—whole abdomen with contrast, coronal view (12 months). **C** Computed tomography—whole abdomen without contrast, coronal view (19 months). **D** Computed tomography—whole abdomen without contrast, sagittal view (19 months). **E** Chest X-ray, AP (19 months)

To evaluate the patient, a routine general CT scan was scheduled every 6 months. The first follow-up showed no malignancy residue sign in the operation area and no suspicion of metastasis in lymphatic nodes or other organs, as seen by a PET scan a month after the first surgery. Then, after 6 months, the patient was reassessed, and local recurrence was found. There are multiple new calcified nodules at the left renal bed and calcified lymphadenopathy in paraaortic-aortocaval groups. He was managed by radical re-excision of the tumor and optimized quality of life-on the basis of patient complaints. However, on the third follow-up, the mass became more extensive than the last imaging, and there were more calcified nodules in the renal beda, hepatogastric area, and lymph nodes. The patient was diagnosed with residual extraskeletal osteosarcoma and a biopsy was performed. He survived 20 months after diagnosis with 6 months of progression-free survival. He died 2 weeks after we found evidence of lung and peritoneal metastases (Fig. 5).

## Discussion

Extraskeletal osteosarcoma is a high-grade spindle cell tumor, usually more than 5 cm in size. Jenson *et al*. reported 18/25 cases located at intramuscular [1]. As a scarce case, patients with ESOS commonly come with chief complaints of soft tissue mass that grows slowly. The duration of the symptoms is about 4–6 months, with 50% of them feeling pain in the area of the lesion [1, 6].

History of radiation exposure and trauma were associated as risk factors for developing ESOS, where around 10% of the cases have prior radiation exposure. Laskin *et al*. reported that 13% of all sarcoma cases are radiation-related ESOS. On the other hand, a history of trauma is found in 12–30% of cases, which becomes essential data in managing patients. Trauma history may lead to myositis ossificans as one of the differential diagnoses of ESOS. Furthermore, there is a literature report on osteosarcoma development from myositis [1, 6].

In managing tumor lesions, a proper history and physical findings are crucial. However, a radiology examination must be done to get an appropriate diagnosis. For initial imaging studies, plain radiography can show soft tissue tumors with massive or dot calcification. Magnetic resonance imaging (MRI) helps determine the local staging and gives adjunct information for preoperative preparation before resecting the tumor in an operable case. Computed tomography (CT) plays a role in identifying mineralization and necrosis in the tumor and in recognizing bone involvement or metastases. Bone scintigraphy and positron emission tomography (PET) scans help determine the tumor stage [7, 8].

On the basis of history-taking and physical examination of the patient, supported by abdominal USG results, it was suggested as a nonmalignancy case. However, an abdominal CT without contrast and a CT urography with contrast revealed a left retroperitoneal tumor-infiltrating the pancreas, spleen, and left kidney. These odds may happen because the accuracy of history-taking depends on the patient’s awareness and memorization. The symptoms in malignancy cases are also nonspecific or asymptomatic in the early stages, contributing to early diagnostic challenges. Ultrasonography is highly operator-dependent, so it is crucial to consider the other diagnostic possibilities on the basis of the surgeon’s clinical judgment [9].

From our clinical assessment and CT results, a laparotomy was performed as the following management. We found a tumor that infiltrated the pancreas, spleen, gastric region, and left kidney. Thus, the tumor was resected, followed by distal pancreatectomy, partial gastrectomy, splenectomy, and left nephrectomy. Excluding all possible differential diagnoses on the basis of the lesion’s demographics, location, and presentation, and radiology also plays a crucial role [1]. Some other tumor-like lesions mimic ESOS, such as osteosarcoma with tumoral osteoid production, reactive metaplastic bone, dense collagen-mimicking osteoid, or even a benign soft tissue tumor with bone formation. Especially in this case, where the tumor was located at the retroperitoneal, dedifferentiated liposarcoma is a more prevalent entity possibly happening [10]. All specimens were sent for histological examination, which revealed high-grade, pleomorphic, vesicular, chromatin tumor cells with spindle nuclei and involvement of cartilage and osteoid matrix that forms a lace-like pattern. These findings suggest osteosarcoma as the diagnosis. The scarcity of ESOS results in diagnostic difficulties owing to radiological and pathological similarities with other conditions, which are more common [10]. Making a correct diagnosis of ESOS is vital because it needs a more aggressive treatment strategy than other sarcomas [11].

There are no typical IHC characteristics of ESOS. However, an immunohistochemistry examination can help make a suggestive diagnosis and exclude other possibilities. Osteoblastic EOS may express nuclear *SATB2*, but this does not confirm a diagnosis of EOS because other tumors with tumoral bone focal may also show *SATB2* expression. In some conditions, ESOS may express *SMA*, *S-100*, and cytokeratins in a weak and patchy area. The findings of genetic protein expression in ESOS are relatively nonspecific. Around 10–20% of cases may show* MDM2* and *CDK4 *amplification, which is typically found in dedifferentiated liposarcoma. Deletions of *H3K27me3* may occur in ESOS but are more commonly found in MPNST with bone involvement. If there is an *H3K27me3* deletion, we should look for *SOX10* and expression, and if none, then it is ESOS.

Our institution has a sufficient IHC panel to exclude another variant of tumor type and make a diagnosis of ESOS. We found positive expression of *SATB2*, which is common in ESOS cases. A differential diagnosis of MPNST becomes unlikely because there is no expression of *S-100* from our IHC examination. Other possibilities, such as dedifferentiated liposarcoma and fibrosarcoma, can be excluded because there is no lipid component or fibroblast proliferation from the hematoxylin–eosin examination. On the basis of histopathology typical osteosarcoma findings and the exclusion of other possibilities from IHC examination, we made a final diagnosis of a rare extraskeletal osteosarcoma in the patient.

This rare soft-tissue tumor is associated with significantly poor morbidity and mortality owing to the lack of definitive proper treatment guidelines available. The best treatment approach has not been established with the limited cases reported. Some clinicians suggest treating these cases as high-risk soft tissue sarcoma, while some prefer to treat them as conventional osteosarcoma. With the conventional approach for ESOS, Paludo *et al*. report an overall objective response (27%) using preoperative platinum-based chemotherapy without a significant survival advantage compared with non platinum therapy or receiving chemotherapy [11]. In contrast, some outmoded cohort studies reported 25% versus 66% 5-year overall survival, in which chemotherapy was superior [12, 13]. However, Nystrom *et al*. reported that the 5-year overall survival was only 11.7%, with an increase in the survival length in patients who received chemotherapy (16.4 versus 9.3 months, *p* = 0.16) [14]. The management approach for ESOS depends on the clinical analysis and judgment, where most of them use soft tissue sarcoma guidelines (anthracycline with or without ifosfamide) or primary osseous sarcoma (cisplatin, doxorubicin, ifosfamide, and methotrexate). However, adjuvant multiagent chemotherapy shows a nonstatistical superiority to improve disease-free survival of the patient and is not recommended for routine use [14, 15].

This case showed a large nonmetastatic disease at diagnosis in an elderly patient, followed by recurrence of the tumor lesion after radical resection 6 six months and metastatic progression after 19 months. Some of these findings are poor prognostic markers, which may be the answer for the survival outcomes for the patient [11]. The recurrence rate of ESOS reaches 45–50% within 6–9 months after surgery, similar to our case after 7 months. The patient showed peritoneal and pulmonary metastasis after treatment; the pulmonary site is the most common metastatic area with a 62–65% rate [14].

## Conclusion

This case report demonstrates the complex diagnostic process of extraskeletal osteosarcoma, which requires a multimodal examination. Histological examination can suggest the diagnosis of ESOS with high-grade pleomorphic spindle cells and osteoid matrix involvement. Owing to the rarity of ESOS and overlapping features with other conditions, immunohistochemistry (IHC) is needed to make a definitive diagnosis. *SATB2* positivity suggested ESOS, while *S-100* negativity ruled out other possibilities. Treatment often mirrors that of high-risk soft tissue sarcomas or conventional osteosarcomas, with varying chemotherapy regimens showing limited survival benefits. In this case, the patient experienced recurrence and metastasis, highlighting the aggressive nature and poor prognosis associated with ESOS.

## Data Availability

Data sharing is not applicable to this article as no datasets were generated or analyzed during the current study.

## References

[1] Kattepur AK, Gulia A, Jones RL, Rastogi S (2021). Extraskeletal osteosarcomas: current update. Future Oncol.

[2] Hoch M, Ali S, Agrawal S, Wang C, Khurana JS (2013). Extraskeletal osteosarcoma: a case report and review of the literature. J Radiol Case Rep.

[3] Wang H, Miao R, Jacobson A, Harmon D, Choy E, Hornicek F (2018). Extraskeletal osteosarcoma: a large series treated at a single institution. Rare Tumors.

[4] Lee JS, Fetsch JF, Wasdhal DA, Lee BP, Pritchard DJ, Nascimento AG (1995). A review of 40 patients with extraskeletal osteosarcoma. Cancer.

[5] Liao Z, Qiu M, Yang J, Yang Y, Zhu L, Yang B (2019). Outcomes of surgery and/or combination chemotherapy for extraskeletal osteosarcoma: a single-center retrospective study from China. Sci Rep.

[6] Mc Auley G, Jagannathan J, O’Regan K, Krajewski KM, Hornick JL, Butrynski J (2012). Extraskeletal osteosarcoma: spectrum of imaging findings. Am J Roentgenol.

[7] Mudgal P, Kang O. Extraskeletal osteosarcoma. Radiopaediaorg. 2018. https://radiopaedia.org/articles/27393.

[8] Gulia A, Puri A, Jain S, Rekhi B, Juvekar S (2013). Extraskeletal osteosarcoma with synchronous regional lymph node and soft tissue metastasis: a rare presentation of an uncommon tumour. Eur J Orthop Surg Traumatol.

[9] Thomas J, Jerome A, Marr G, De Boo DW, Gani J (2024). Surgeons versus radiologists: do we care what they think?. ANZ J Surg.

[10] Yenwongfai LN, Liu J, Wang C, Bocklage TJ (2022). Extraskeletal osteosarcoma and its histological mimics. Hum Pathol Rep.

[11] Paludo J, Fritchie K, Haddox CL, Rose PS, Arndt CAS, Marks RS (2018). Extraskeletal osteosarcoma: outcomes and the role of chemotherapy. Am J Clin Oncol.

[12] Goldstein-Jackson SY, Gosheger G, Delling G, Berdel WE, Gulrich E, Jundt G (2005). Extraskeletal osteosarcoma has a favorable prognosis when treated like conventional osteosarcoma. J Cancer Res Clin Oncol.

[13] Torigoe T, Yazawa Y, Takagi T, Terakado A, Kurosawa H (2007). Extraskeletal osteosarcoma in Japan: multiinstitutional study of 20 patients from the Japanese Musculoskeletal Oncology Group. J Orthop Sci.

[14] Nystrom LM, Reimer NB, Reith JD, Scarborough MT, Gibbs CP (2016). The treatment and outcomes of extraskeletal osteosarcoma: institutional experience and review of the literature. Iowa Orthop J.

[15] Tsukamoto S, Mavrogenis AF, Angelelli L, Righi A, Filardo G, Kido A (2022). The effect of adjuvant chemotherapy on localized extraskeletal osteosarcoma: a systematic review. Cancers.

